# Retinoblastoma in an adult

**DOI:** 10.1186/1756-0500-6-304

**Published:** 2013-08-01

**Authors:** Saemah Nuzhat Zafar, Saqib Qayyum Ahmad, Nadeem Zafar

**Affiliations:** 1 Department of Pediatric Ophthalmology and Strabismus, Al-Shifa Trust Eye Hospital, Rawalpindi 46000, Pakistan; 2 Department of Pathology, PAF Hospital, Islamabad, Pakistan; 3 Armed Forces Hospital, Najran, Kingdom of Saudi Arabia

**Keywords:** Retinoblastoma, Neovascular, Glaucoma, Ultrasonography, CT-Scan, Eye enucleation, Histopathology, Cytology, Optic nerve

## Abstract

**Background:**

Retinoblastoma is the most common pediatric ocular tumour. It may rarely present in adults. The present case adds to the number of 26 cases already published in literature since 1919 till 2013. Our aim is to highlight the rare occurrence of retinoblastoma in adults along with its features which differentiate it from paediatric retinoblastoma.

**Case presentation:**

We describe a case of adult onset retinoblastoma (group E, according to the international classification of retinoblastoma) occurring in a 25 year old male. He presented with decreasing visual acuity in the right eye of 4 months duration. He had neo-vascular glaucoma and pseudohypopyon. B scan ultrsonography of his right eye showed intraocular growth without any calcification. The CT scan of the orbits and brain showed intraocular growth in the right eye with no calcification. Enucleation of the right eye was carried out. Retinoblastoma was confirmed on histopathology of the enuleated globe.

**Conclusions:**

The present case adds to the number of adult Rb patients reported in literature. Early detection to salvage the life can be made possible if the clinician keeps a high index of suspicion when observing retinal mass of adult onset. Proper counselling of the patient in order to seek his full involvement in management may help in improving the prognosis of the disease.

## Background

Retinoblastoma (Rb) is the most common paediatric ocular tumour. It may rarely present in adults. Only 26 adult cases of Rb have been published in literature till 2013 [1-3]. Because of the rarity of the disease the diagnosis is occasionally delayed. Our aim is to highlight the rarity of occurrence of Rb in adults along with its features which differentiate it from paediatric Rb. We also aim to emphasize the need to suspect Rb in adults, who present with a retinal mass, to ensure its early diagnosis.

## Case presentation

A 25 year old male patient presented to our tertiary care eye hospital with a complaint of decreasing visual acuity in the right eye of 4 months duration. He had been treated earlier with topical anti-glaucoma agents and steroids given orally, topically and as posterior subtenon injection. His previous clinical record showed anterior chamber activity, severe vitritis and an intraocular pressure of 40 mmHg. Intraocular growth had been suspected on a previous B-scan ultrasonography. Vitreous tap of the right eye performed before the patient came to our clinic had shown atypical cells on cytological examination, suggestive of retinoblastoma (Rb).

**Figure 1:**
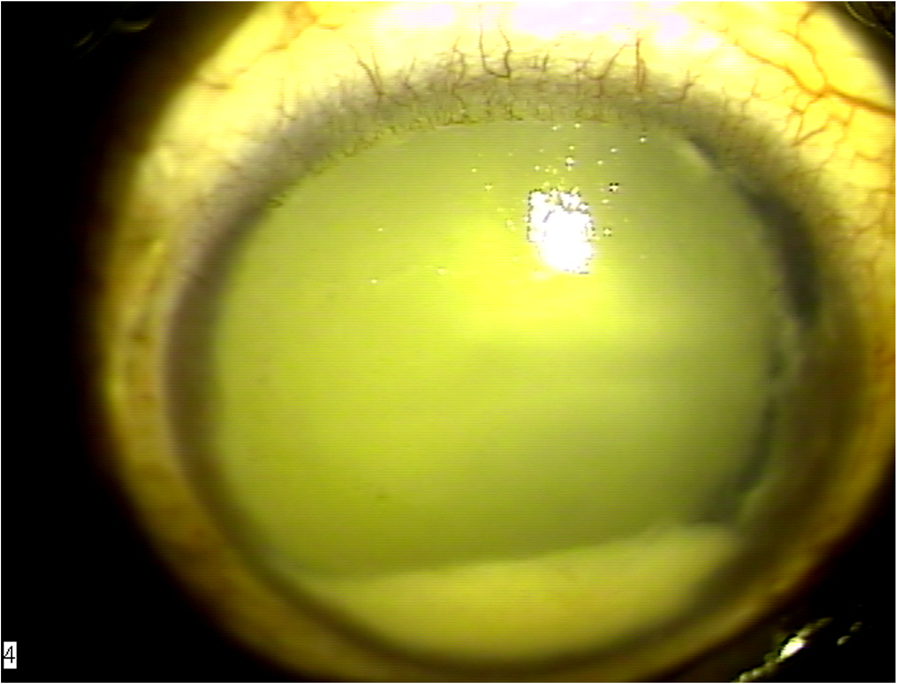
Pseudohypopyon.

On ocular examination he had no perception of light in the right eye (PL –) and 6/6 vision in the left eye. There was rubeosis iridis, ectropion uveae, a fixed pupil and neovascular glaucoma in the right eye. By that time he had also developed a pseudohypopyon in the right eye resembling a masquerade syndrome (Figure 1). Vitritis and vitreous condensations were noticed. The view of the right fundus was not clear. Examination of the left eye did not show any abnormality in the anterior and posterior segments.

**Figure 2:**
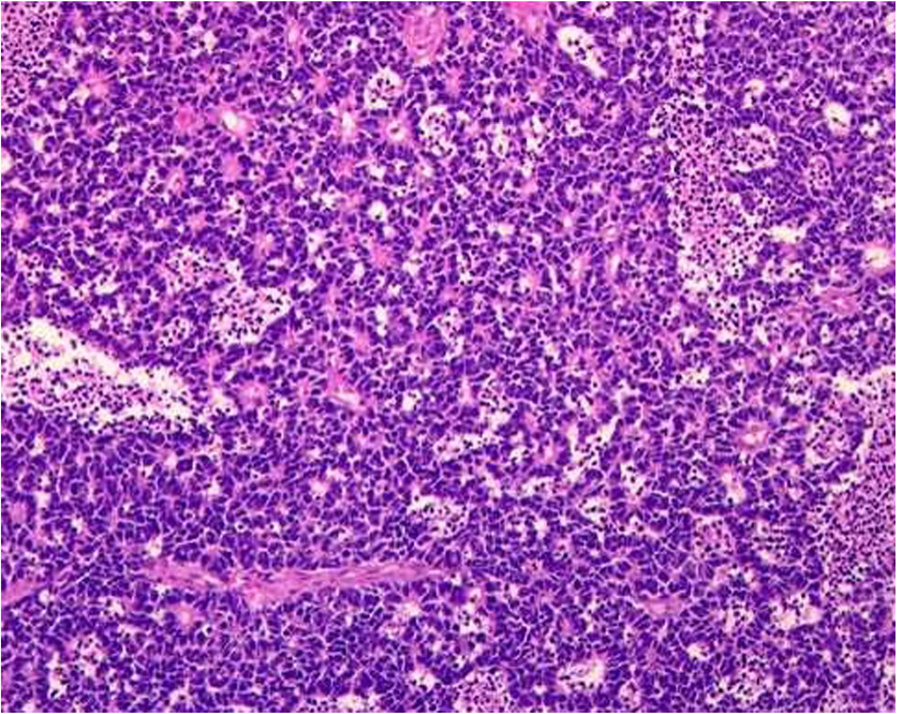
**Photomicrograph showing retinoblastoma, H&E (×100).** The tumour is composed of small undifferentiated cells that also form rosettes.

B-scan ultrasonography of the right eye showed a hyper echoic endophytic mass with no calcification. CT scan of the orbits and brain showed intraocular mass in the right eye without any calcification. The patient had normal blood counts and liver function tests. Enucleation of the right eye was carried out. Histopathological examination revealed poorly differentiated Rb (Figure 2), showing combined endophytic and exophytic growth patterns and retinal detachment. Tumour size was 1.5 × 1.2 cm. Vitreous, choroid and optic disc were involved. Optic nerve was involved up to the resection margin (pT4). Regional lymph nodes were not assessed (pNX). Tumour was poorly differentiated (histological grade pG3) with necrosis greater than 50%. Apoptosis and calcification were also seen on histopathology.

Chemotherapy and radiotherapy were planned and pre-chemotherapy systemic laboratory workup was advised. However, the patient did not comply and was lost to follow up. He reported again after one month with swelling in the enucleated socket. Patient was again counselled about the disease and management with chemotherapy and radiotherapy was advised. Injection Carboplatin 975 mg (on day 1), injection Etoposide 260 mg (on day 1and 2) and injection Vincristine 2 mg (on day 1) were given 4 weekly. After 6 cycles of chemotherapy and radiotherapy of 50 Gy in 25 fractions (12 MeV) to the right orbit, the patient developed sudden weakness of lower limbs with inability to sit or stand. Bone scan was performed using Technetium (^99m^Tc) medronic acid but whole body imaging did not show any evidence of secondaries. DEXA scan of the lumbar spine and left hip showed osteopenia and increased risk of fractures. Contrast enhanced CT scan of the orbits and brain showed a metastatic brain deposit as an enhancing soft tissue mass in the supra-sellar region. Whole brain Co 60 radiotherapy in a dose of 20 Gy in 5 fractions was done. He was then discharged from the oncology unit with an advice to continue supportive care as he was unfit for any further oncology related therapeutic intervention. The patient expired almost 16 months after the onset of symptoms.

## Discussion

Adult onset Rb is very rare therefore it is usually missed in the differential diagnosis of a retinal growth in this age. It is not well established whether Rb in an adult occurs *de novo* or is preceded by a retinocytoma [4]. More than 2 dozen Rb patients older than 20 years of age have been reported in literature. Singh *et al.* gave a review of 24 cases till 2011. Khetan *et al.* and Zhang *et al.* also described one case each in 2012. All were sporadic and like our patient, unilateral [1-3].

The diagnosis of Rb should be considered in adult patients if a whitish mass is seen on fundus examination [4,5]. Rb in adults differs from that in the paediatric age group in being less common. It may mimic Coat’s disease in the adults. Ocular inflammation, vitreous haemorrhage and cataract are more commonly associated adult Rb as compared to its paediatric counterpart. Moreover B-scan ultrasonography may not show any calcification which is more commonly seen in paediatric Rb. All these factors may lead to difficulty in diagnosis [4].

Our patient also had a vitreous tap before enucleation. There is a possibility of upstaging of retinoblastoma due to this procedure. If vitrectomy or vitreous tap is performed in an eye with unsuspected Rb, enucleation combined with adjuvant chemotherapy, radiotherapy, or both without delay is advised to prevent systemic tumour dissemination [6]. Our patient was treated with carboplatin, vincristine and etoposide as a three drug regimen which is the current protocol practiced in many institutions [7].

Ophthalmologists mainly come across Rb in paediatric age group where the prognosis of the disease is good if treatment is started at an early stage. In the adults the prognosis is worse therefore patients must be counselled regarding adherence to the treatment. Our patient had remained lost to follow up till one month after enucleation. This fact shows that the counselling, monitory help, and liaison with oncology services should be made more effective and coordinated, especially in developing countries, where various socioeconomic factors lead to non-compliance by the patients.

## Conclusion

The present case adds to the number of adult Rb patients reported in literature. Early detection to salvage the life can be made possible if the clinician keeps a high index of suspicion when observing retinal mass of adult onset. Proper counselling of the patient in order to seek his full involvement in management may help in improving the prognosis of the disease.

## Consent

Written informed consent was obtained from the patient for publication of this Case Report and accompanying images. A copy of the written consent is available for review by the Editor-in-Chief of this journal.

## Competing interests

The authors declare that they have no competing interests.

## Authors’ contributions

All authors have made substantial contributions to all of the following: (1) the conception and design of the study, or acquisition of data, analysis and interpretation of data, (2) drafting the article and revising it critically for important intellectual content, (3) final approval of the version to be submitted. All authors read and approved the final manuscript.
